# Increased ANGPTL3, 4 and ANGPTL8/betatrophin expression levels in obesity and T2D

**DOI:** 10.1186/s12944-016-0337-x

**Published:** 2016-10-13

**Authors:** Mohamed Abu-Farha, Irina Al-Khairi, Preethi Cherian, Betty Chandy, Devarajan Sriraman, Asma Alhubail, Faisal Al-Refaei, Abdulmohsen AlTerki, Jehad Abubaker

**Affiliations:** 1 Biochemistry and Molecular Biology Unit, Dasman Diabetes Institute, P.O. Box 1180, Dasman, 15462 Kuwait; 2 Tissue Banking Unit, Dasman Diabetes Institute, Kuwait City, Kuwait; 3 Clinical Services Department, Dasman Diabetes Institute, Kuwait City, Kuwait

## Abstract

**Background:**

Hypertriglyceridemia is associated with increased risk for cardiovascular diseases and type 2 diabetes (T2D). Angiopoietin like proteins particularly 3, 4 and recently 8 are well established regulators of plasma triglyceride level through regulating the activity of lipoprotein lipase. Plasma level and association between ANGPTL3, 4 and 8 is not well established in human subjects. This study was designed to establish the level of these proteins in plasma and adipose tissues and investigate the association between ANGPTL8 with ANGPTL3 and 4 in T2D and non-diabetics subjects.

**Methods:**

A total of 235 subjects were enrolled in this study, 144 non-diabetics and 91 T2D. ANGPTL 3, 4 and 8 levels were measured in plasma by ELISA and using real time RT-PCR in adipose tissues.

**Results:**

In this study, we showed that ANGPTL3, 4 and 8 were higher in T2D subjects. Dividing the non-diabetic subjects according to their BMI showed higher level of ANGPTL3, 4 and 8 in obese subjects compared to non-obese subjects. No significant difference was observed between the T2D subjects. ANGPTL8 was showed positive correlation with ANGPTL3 in the non-diabetic subjects in the non-obese (*r* = 0.2437, *p*-Value = 0.0543) and obese subjects (*r* = 0.418, *p*-Value = 0.0125). No association was observed in the T2D subjects. On the other hand, ANGPTL4 was positively associated with the obese subjects in both the non-diabetics (*r* = 0.3322, *p*-Value = 0.0316) and the obese T2D subjects (*r* = 0.3161, *p*-Value = 0.0211).

**Conclusion:**

In conclusion, our data shows that ANGPTL3, 4 and 8 are increased in obesity and T2D. ANGPTL8 associates with ANGPTL3 in the non-diabetic subjects while it associated more with ANGPTL4 in the obese and T2D subjects. Taken together, this data highlight the role of these proteins in metabolic diseases and how they interact with each other’s under different physiological and pathophysiological conditions.

## Background

Hypertriglyceridemia has been linked to increased incidents of cardiovascular disease and Type 2 diabetes (T2D) [1, 2]. Triglycerides (TG) are essential to human life as they provide energy and store excess energy in the body [3, 4]. They circulate in blood as lipoprotein molecules such as chylomicrons that are formed from dietary TG or VLDL, which are produced by the liver under fasting state [5, 6]. Chylomicrons and VLDL mediate the transfer of TG to various tissues for storage or oxidation to produce energy [3, 4]. The key enzyme involved in the breakdown of TG to generate free fatty acids (FFA) is lipoprotein lipase (LPL) [7, 8]. LPL activity regulates TG clearance and its availability for other tissues such as heart and muscle. LPL hyperactivity has been associated with decreased plasma TG level and decreased cardiovascular risks while its loss of function resulted in severe hypertriglyceridemia [7, 8]. As a result its cellular function is tightly regulated to accommodate the need of various tissues for TG and to maintain physiological TG blood level [7, 8].

One of the key regulators of LPL are members of the angiopoietin-like protein (ANGPTL) family mainly ANGPTL3, 4 and 8 [7, 8]. Seven members have been traditionally assigned to the ANGPTL family due to their similarity to angiopoietin proteins [9, 10]. All members have N-terminal coiled-coil domain as well as a carboxyl-terminal fibrinogen-like domain, except the recently added ANGPTL8 family member which lacks the fibrinogen-like domain [9–12]. The role of ANGPTL3 and 4 in regulating LPL activity has been well established. ANGPTL3 is produced by the liver then it is proteolytically cleaved by proprotein convertases to generate an active N-terminal domain that acts as an inhibitor of LPL activity [12, 13]. One study showed that loss of function mutations identified in ANGPTL3 associated with decreased VLDL, LDL, HDL and TG [14]. This was also linked to a recessive disorder disease termed familial combined hypobetalipoproteinemia [14, 15]. ANGPTL4 is another potent inhibitor of LPL activity that is induced in the fasting state by the PPAR (peroxisome proliferator-activated receptor) transcription factor [7]. Similar to ANGPTL3, the N-terminal domain of ANGPTL4 is cleaved by proprotein convertases and act as an inhibitor of LPL activity [7]. The inhibition of LPL activity occurs through the direct binding of the cleaved ANGPTL4 to LPL protein, which in turn inhibits its dimerization rendering the LPL enzyme inactive [8, 16]. Recent data has also linked loss of function mutations in ANGPTL4 to reduced incidences of Coronary heart disease [17, 18]. Due to their role in regulating LPL activity and the fact that many genome wide association studies has linked them to dyslipidemia, ANGPTL3 and 4 are being explored as drug targets for metabolic diseases [16]. Specific monoclonal antibodies targeting ANGPTL3 and 4 has been shown to decrease plasma lipid content [16].

ANGPTL8 on the other hand has been shown to regulate LPL activity through its interaction with the N-terminal domain of ANGPTL3, which facilitates its cleavage [12, 19]. ANGPTL8 is mainly expressed in the liver, white adipose tissue as well as brown adipose tissue [12, 19–21]. It has been initially shown to be induced by feeding and reduced by fasting [20, 21]. Similar to ANGPTL3 and 4, sequence variation in ANGPTL8 has been associated with decreased LDL and HDL lipid profile [12]. Quagliarini et al. showed that rs2278426, which represents a non-synonymous amino acid change from Arginine (R) to tryptophan (W) at amino acid 59, was associated with lower LDL-C and HDL-C in various ethnic groups [12]. We have also showed that this variant was associated with higher plasma glucose in Arabs [12]. Collectively, ANGPTL3, 4 and 8 are shown to play an important role in the regulation of LPL activity. Therefore, further research on the collective role of these three proteins in humans is critical to enhance our understanding of lipid metabolism. As a result we designed this study to investigate the possible changes in the expression levels in circulation and adipose tissue in both diabetic and non-diabetic subjects. In addition, we investigated the possible association between ANGPTL8 and ANGPTL3 and 4 in this population to better understand their physiological role.

## Methods

### Study population and ethical statement

In the present study, a total of 144 non-diabetics and 91 T2D subjects were recruited at Dasman Diabetes Institute. Based on the Body Mass Index (BMI) the non-diabetic subjects were split into a total of 82 non-obese subjects (19.5 and ≤ BMI < 30 kg/m^2^) and 62 obese subjects (30 ≤ BMI < 40 kg/m^2^). On the other hand, 27 T2D subjects were classified as non-obese and 64 were obese. The formula used to calculate BMI is the standard BMI formula: body weight (in kilograms)/height (in meters squared). All subjects signed a written informed consent before their participation in the study, which was approved by the Ethical Review Board of Dasman Diabetes Institute and abiding with the guideline ethical declaration of Helsinki. Morbidly obese subjects (BMI >40 kg/m^2^) as well as subjects with prior major illness or taking any medication and/or supplement known to influence the body composition or bone mass were excluded from the study [22].

### Blood collection, anthropometric and biochemical measurements

Blood samples were collected in vacutainer EDTA tubes that were centrifuged to collect plasma samples which in turn were aliquoted and stored at −80 °C until assayed as described previously [22–24]. Subcutaneous adipose tissue (SAT) biopsies were obtained from the periumbilical area by surgical biopsy after a local anaesthesia as described previously [22]. Following the surgical extraction of SAT tissues they were rinsed in cold PBS and stored at −80 °C in Allprep until assayed.

The anthropometric and biochemical measurements were collected as follows: an average of three blood pressure readings measured using an Omron HEM-907XL Digital sphygmomanometer were taken with a 5–10 min rest between each reading. Whole-body composition was determined by dual-energy radiographic absorptiometry device (Lunar DPX, Lunar radiation, Madison, WI). Fasting blood Glucose (FBG) triglyceride (TG), total cholesterol (TC), low density lipoprotein (LDL) and high density lipoprotein (HDL) were measured on the Siemens Dimension RXL chemistry analyzer (Diamond Diagnostics, Holliston, MA). Glycated hemoglobin (HbA1C) was determined using the Variant^TM^ device (BioRad, Hercules, CA).

### ANGPTL8 ELISA

ANGPTL8 level was measured in plasma as described previously [23, 25, 26]. Plasma samples were thawed on ice and centrifuged at 10000xg for 5 min at 4 °C to remove any debris as described previously [22–24]. Repeated freeze thaw cycles were avoided. ANGPTL8 level was determined using the ELISA kit from Wuhan EIAAB Science co (catalogue number E1164H) as described previously [23, 25, 26]. No significant cross reactivity with other proteins was observed. Intra-assay coefficients of variation (CV) were 2.1 to 4.6 %, while the inter-assay coefficients of variation were 7.3 to 9.6 %.

### ANGPTL3 and 4 ELISA

Plasma levels of ANGPTL3 and 4 were assessed using the multiplexing immunobead array platform according to the manufacturer instructions (R & D systems). The data was processed using the Bio-Plex manager software version 6 (Bio-Rad) using five-parametric curve fitting. Intra plate CV ranged from 7.0 to 12 %, while inter plate CV was <14 %. Samples were measured using reagents from the same lot to avoid lot- to lot variations.

### Measurement of gene expression by quantitative Real Time-PCR (qRT-PCR)

Total RNA was extracted from frozen SAT tissues using RNeasy Lipid Tissue Mini Kit according to manufacturer’s protocol (Qiagen, Inc., Valencia, CA). Total RNA was isolated from adipose tissue biopsies of obese non-diabetic (*n* = 8) and obese diabetic (*n* = 8). The cDNA was prepared from total RNA sample using High Capacity cDNA Reverse Transcription Kits (Applied Biosystems, Foster City, CA). QRT-PCR was performed on Rotor-Disc 100 system using SYBR Green normalized to Gapdh (Qiagen, Inc., Valencia, CA). The following primer sequences were used for gene expression quantification: ANGPTL3 forward: TCTCCAGAGCCAAAATCAAGAT and Reverse: TTTCACTGGTTTGCAGCGAT. ANGPTL4 forward: CAGTCCTCGCACCTGGAA and reverse: GCCAGGACATTCATCTCGTC. ANGPTL8 forward: AATCTGCCTGGATGGAACTG and reverse: CTGCGTCTGTCTCTGCTCTG. GAPDH forward: AACTTTGGCATTGTGGAAGG-3′ and reverse: TGTGAGGGAGATGCTCAGTG. Relative expression was assessed by using the ∆∆CT method [27].

### Statistical analysis

Comparisons between non-obese and obese subjects were made by Student’s *t*-test. Spearman’s correlation coefficients were estimated to determine associations of ANGPTL8 with ANGPTL3 and/or 4. All data are reported as mean ± standard deviations. Statistical assessments were two-sided and considered to be significant when *p*-Value < 0.05. All analyses were performed using SAS (version 9.2; SAS Institute).

## Results

### Study population characteristics

Clinical and biochemical characteristics of the non-diabetics and T2D subjects are shown in Table 1. Our population was made of 235 subjects divided into 144 non-diabetic and 91 T2D subjects. The average age of the non-diabetic subjects was 41.76 ± 11.81 years while the average age of T2D subjects were 52.89 ± 9.32 years. T2D subjects had significantly higher BMI, percent body fat, TG, TC, HDL, FBG, and HbA1C (*p*-Value < 0.05) (Table 1). T2D subjects had significantly higher level of ANGPTL8 (2520.14 ± 155.31 pg/ml) compared to the non-diabetics (936.78 ± 55.43 pg/ml, *p*-Value < 0.001). ANGPTL4 was also higher in T2D subjects (203.78 ± 11.68 ng/ml compared to 144.47 ± 4.47 ng/ml for the non-diabetics) (*p*-Value < 0.001). A similar trend was also observed for ANGPTL3 in T2D 69.17 ± 3.07 ng/ml and 62.39 ± 1.89 ng/ml for the non-diabetics (*p*-Value = 0.06) Table 1.

**Table 1:** Characteristics of the non-diabetic and diabetic subjects included in this study

Variable	Average ± SD Non-Diabetics *N* = 144	Average ± SD T2D *N* = 91	*P*-Value
Age (years)	41.76 ± 11.81	52.89 ± 9.32	<0.000
BMI (kg/m2)	29.06 ± 5.87	31.64 ± 4.30	<0.000
Percent Body Fat	34.56 ± 6.68	36.27 ± 5.56	0.023
TC (mmol/L)	5.07 ± 0.93	4.94 ± 1.29	0.282
HDL (mmol/L)	1.27 ± 0.38	1.19 ± 0.46	0.087
LDL (mmol/L)	3.20 ± 0.86	3.02 ± 1.26	0.110
TG (mmol/L)	1.29 ± 0.90	1.87 ± 2.18	0.003
FBG (mmol/L)	5.32 ± 1.01	8.64 ± 3.31	<0.000
HbA1C (DCCT%)	5.67 ± 0.85	7.98 ± 1.85	<0.000
ANGPTL8 pg/ml	936.78 ± 55.43	2520.14 ± 155.31	<0.000
ANGPTL4 ng/ml	144.47 ± 4.47	203.78 ± 11.68	<0.000
ANGPTL3 ng/ml	62.39 ± 1.89	69.17 ± 3.07	0.062

### Plasma level of ANGPTL3, 4 and 8 in the whole population

**Fig. 1:**
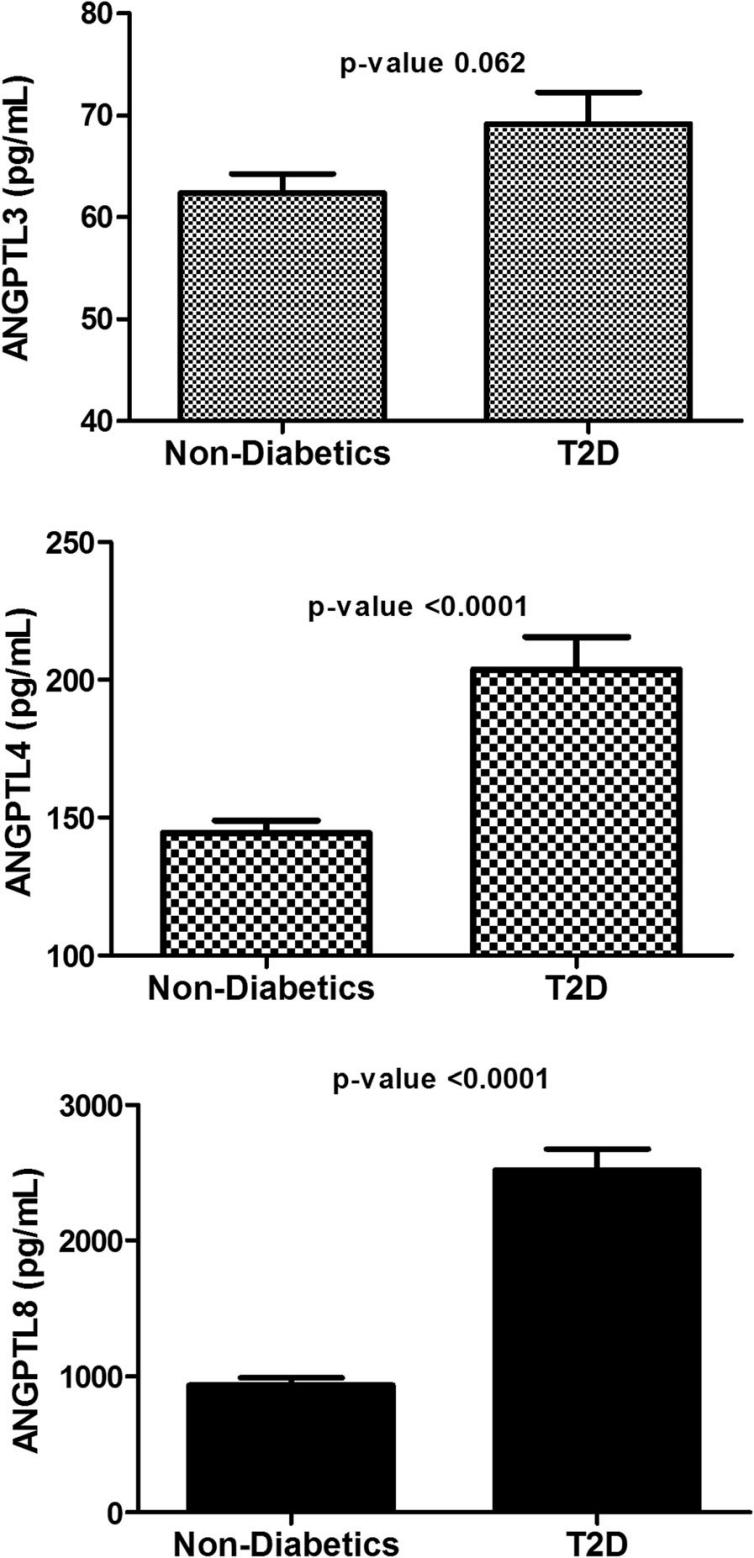
Circulation level of ANGPTL3, 4 and 8 in all subjects measured by ELISA. **a** Circulation level of ANGPTL3 in non-diabetic vs. T2D subjects in all subjects. **b** Plasma level of ANGPTL4 in non-diabetic vs. T2D subjects in all subjects. **c** Circulation level of ANGPTL8 in non-diabetic vs. T2D subjects in all subjects

Plasma level of all markers was examined between non-diabetic and T2D subjects. Overall, plasma level of ANGPTL3, 4 and 8 was higher in the T2D subjects. ANGPTL3 plasma level in the non-diabetic subjects was 62.39 ± 1.89 ng/mL compared to 69.17 ± 3.07 in the T2D subjects (*p*-Value = 0.062) Fig. 1a. ANGPTL4 plasma level was 144.47 ± 4.47 ng/mL in the non-diabetic subjects vs 203.78 ± 11.68 ng/mL in the T2D subjects (*p*-Value <0.0001) Fig. 1b. Finally, ANGPTL8 level was also higher in the T2D subjects 2520.14 ± 155.31 pg/mL compared to 936.78 ± 55.43 pg/mL in the non-diabetic subjects (*p*-Value <0.0001) Fig. 1c.

### Plasma level of ANGPTL3, 4 and 8 in T2D subjects

**Fig. 2:**
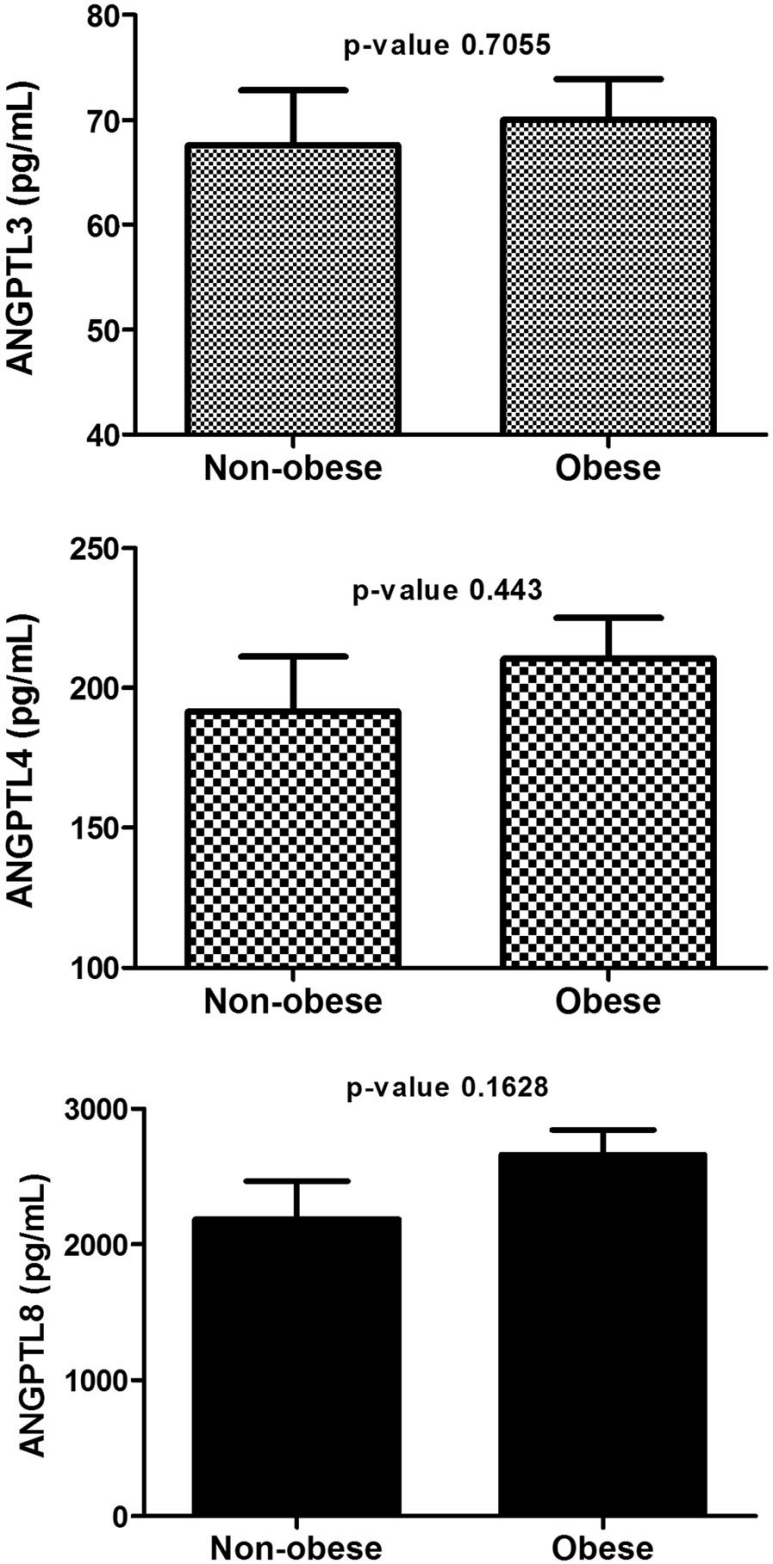
Circulation level of ANGPTL3, 4 and 8 T2D subjects in accordance to their obesity level measured by ELISA. **a** Circulation level of ANGPTL3 in non-obese vs obese T2D subjects. **b** Plasma level of ANGPTL4 in non-obese vs obese T2D subjects. **c** Plasma level of ANGPTL8 measured by ELISA T2D subjects

In the T2D subjects, the circulation level of ANGPTL3, 4 and 8 were higher in the obese subjects however, it did not reach statistical significance. ANGPTL3 level in the non-obese subjects was 67.59 ± 5.18 ng/ml compared to 70.04 ± 3.84 ng/ml in the obese subjects (*p*-Value = 0.7055) Fig. 2a. ANGPTL4 level in non-obese subjects was 191.70 ± 19.52 ng/ml vs 210.50 ± 14.62 ng/ml in the obese subjects (*p*-Value =0.443) Fig. 2b. Finally, ANGPTL8 level in the non-obese subjects was 2185.48 ± 283.58 pg/ml compared to 2661.32 ± 184.19 pg/ml for the obese subjects (*p*-Value = 0.1628) Fig. 2c.

### Plasma level of ANGPTL3, 4 and 8 in the non-diabetic subjects

**Fig. 3:**
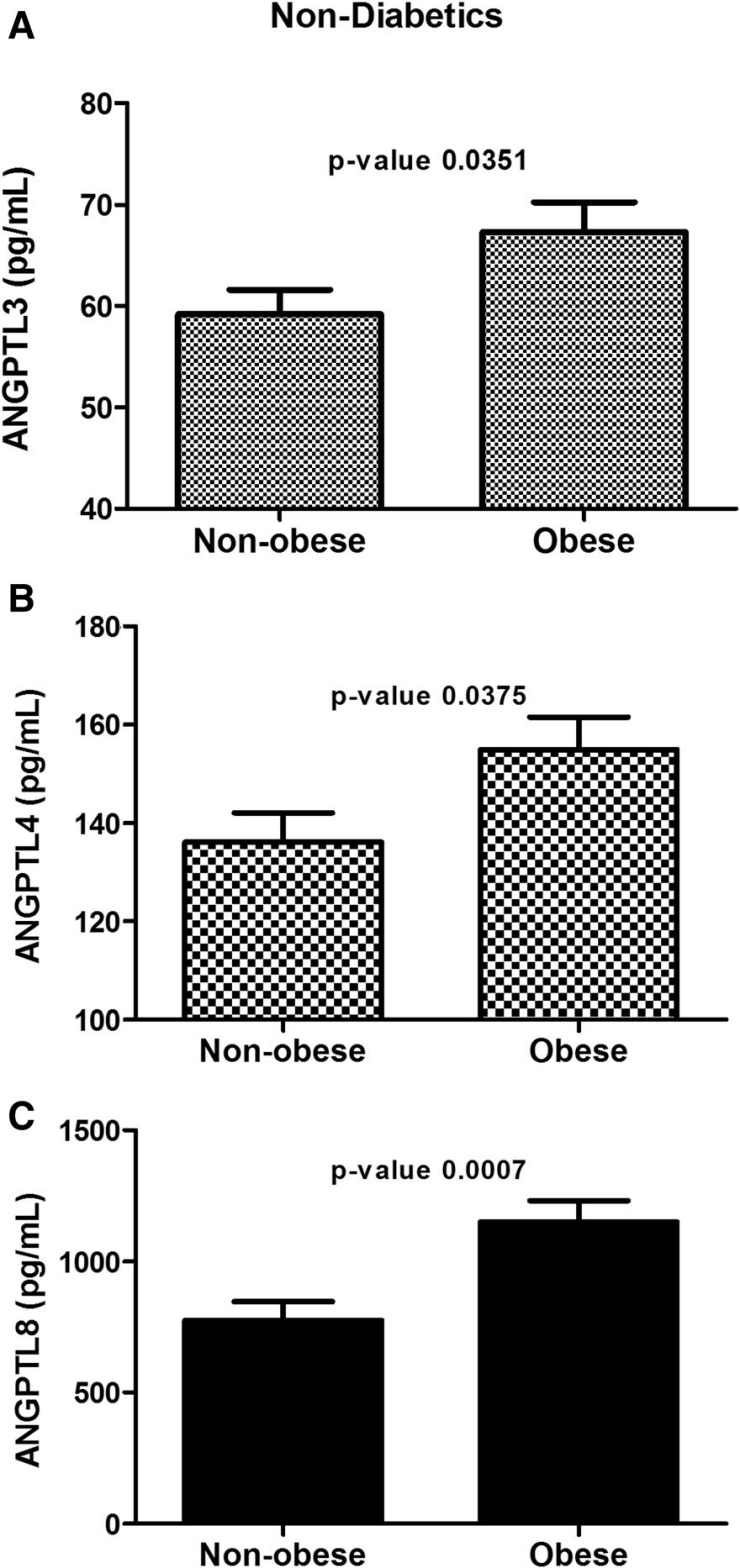
Circulation level of ANGPTL3, 4 and 8 in non-diabetic subjects in accordance to their obesity level measured by ELISA. **a** Circulation level of ANGPTL3 in non-obese vs obese non-diabetic subjects. **b** Circulation level of ANGPTL4 in non-obese vs obese non-diabetic subjects. **c** Plasma level of ANGPTL8 in non-obese vs obese non-diabetic subjects

Overall, plasma level of ANGPTL3, 4 and 8 were higher in T2D subjects compared to non-diabetics as shown in Fig. 1a, b and c. To better understand the possible association of these protein with obesity, the non-diabetics and T2D subjects were further divided into obese and non-obese groups. ANGPTL3 level was significantly higher in the obese subjects compared to the non-obese subjects (67.30 ± 2.96 ng/ml and 59.17 ± 2.40 ng/ml (*p*-Value = 0.0351) Fig. 3a. Similarly, ANGPTL4 was also increased in the obese subjects compared to non-obese (154.88 ± 6.64 ng/ml vs 136.19 ± 5.92 ng/ml respectively, *p*-Value = 0.0375) Fig. 3b. Finally, ANGPTL8 showed a similar trend where its level was higher in the obese subjects (1150.04 ± 81.39 pg/ml compared to 775.54 ± 70.77 pg/ml for the non-diabetics, *p*-Value = 0.0007) Fig. 3c.

### ANGPTL3, 4 and 8 gene expression level in adipose tissue

**Fig. 4:**
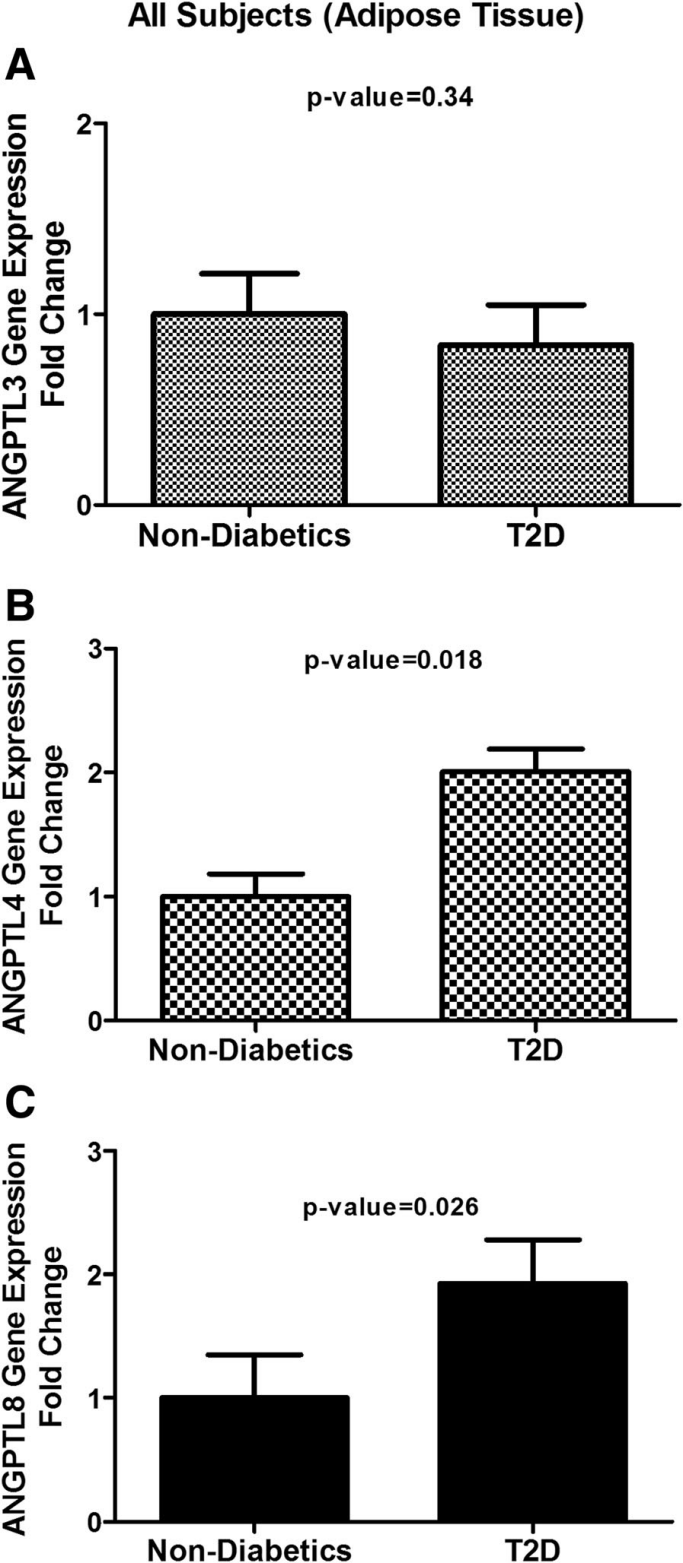
ANGPTL3, 4 and 8 adipose tissue expression level in all subjects. **a** Gene expression level of ANGPTL3 in adipose tissue extracted from non-diabetic and T2D subjects as measured by qRT-PCR. **b** Gene expression level of ANGPTL4 in adipose tissue extracted from non-diabetic and T2D subjects as measured by qRT-PCR. **c** Gene expression level of ANGPTL8 in adipose tissue extracted from non-diabetic and T2D subjects as measured by qRT-PCR

ANGPTL3, 4 and 8 gene expression level in adipose tissue was examined using qRT-PCR. Unlike its plasma level, ANGPTL3 gene expression level was not changing in the adipose tissue extracted from either T2D or non-diabetic subjects (*p*-Value = 0.356) Fig. 4a. However, ANGPTL4 level showed a two fold increase in T2D subjects compared to the non-diabetic subjects as shown in Fig. 4b (*p*-Value = 0.018). Similarly, ANGPTL8 showed around two fold increases in its gene expression level in the T2D subjects compared to the non-diabetic subjects as shown in Fig. 4c (*p*-Value = 0.026).

### Correlation between ANGPTL8 and ANGPTL3

**Fig. 5:**
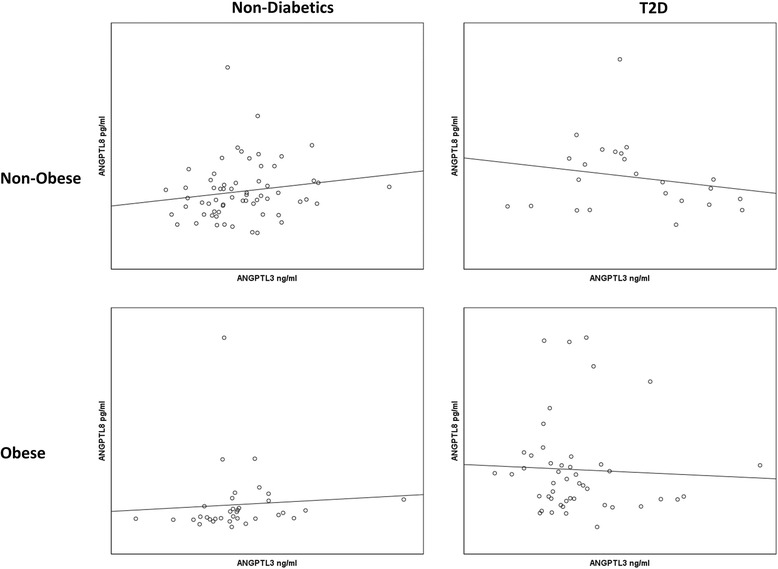
Spearman’s correlation between ANGPTL8 and ANGPTL3 in both non-diabetic and diabetic subjects divided into non-obese and obese subjects according to their BMI

Spearman’s correlation showed that ANGPTL8 associated with ANGPTL3 in the non-diabetics subjects. It showed significant association in the non-obese (*r* = 0.2437, *p*-Value = 0.0543). However, a stronger correlation was observed between ANGPTL8 and ANGPTL3 in the obese subjects (*r* = 0.418, *p*-Value = 0.0125), Fig. 5. On the other hand no association was observed between ANGPTL8 and ANGPTL3 in the T2D subjects for both obese (*r* = −0.1431, *p*-Value = 0.3733) and non-obese groups (*r* = −0.200, *p*-Value = 0.3488) as shown in Fig. 5.

### Correlation between ANGPTL8 and ANGPTL4

**Fig. 6:**
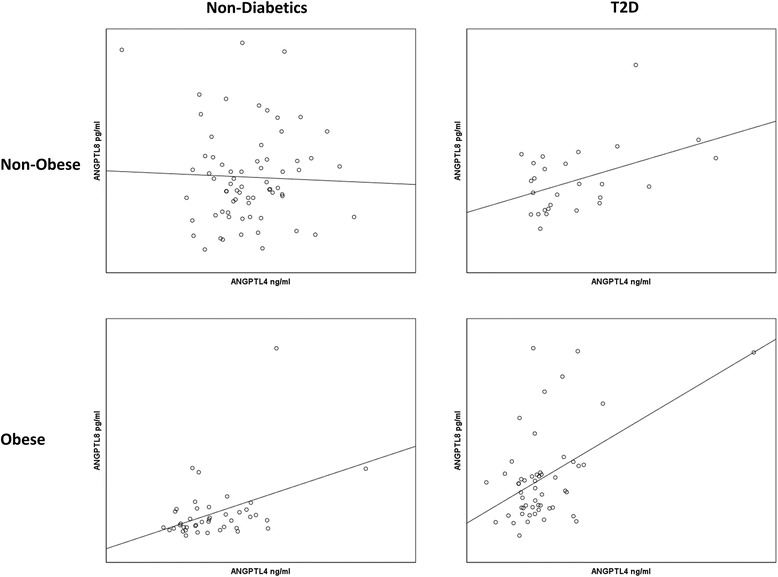
Spearman’s correlation between ANGPTL8 and ANGPTL4 in both non-diabetic and diabetic subjects divided into non-obese and obese subjects according to their BMI

Unlike ANGPTL3, Spearman’s correlation showed no association between ANGPTL8 and ANGPTL4 in the non-diabetics non-obese subjects (*r* = 0.0997, *p*-Value = 0.4186) as well as the non-obese T2D subjects (*r* = 0.3156, *p*-Value = 0.1088) Fig. 6. Nonetheless, it was significantly associated with ANGPTL4 in both obese non-diabetics (*r* = 0.3322, *p*-Value = 0.0316) and obese T2D subjects (*r* = 0.3161, *p*-Value = 0.0211) as shown in Fig. 6.

## Discussion

In order to investigate the relationship between ANGPTL3, 4 and 8, we measured their expression level in plasma and adipose tissue in T2D and non-diabetic subjects in the whole population as well as in obese and non-obese subjects. Overall, plasma level of ANGPTL 3, 4 and 8 were increased in subjects with T2D compared to non-diabetic subjects. Similarly, Obese/non-diabetic subjects showed a significant increase in the plasma expression level of ANGPTL3, 4 and 8 compared to non-obese subjects. On the other hand, no significant difference was observed between the plasma protein levels in obese and non-obese T2D subjects. Similar to the protein level in circulation, adipose tissue ANGPTL4 and 8 gene expression level showed a two fold increase in T2D subject compared to non-diabetics. However, gene expression level of ANGPTL3 was not affected. ANGPTL8 showed significant correlation with ANGPTL3 in both obese and non-obese groups in the non-diabetic subjects. ANGPTL4 on the other hand correlated positively with ANGPTL8 only in the obese subjects from both T2D and non-diabetic subject groups.

The hydrophobic nature of lipids prevent them from circulating freely in blood unless emulsified by proteins forming lipoprotein complexes that circulate the blood stream to function as a source of energy, structural blocks and singling molecules [9, 28–30]. TG is an essential lipid molecule that is used to provide energy for the body [9]. It exists in circulation in the form of chylomicrons and very low density lipoprotein (VLDL). Chylomicrons are formed, after eating, in the villi of the duodenum and secreted in the blood stream while VLDL are formed, in the fasting state, by the liver and released in blood stream [3, 31]. LPL is a key enzyme in the hydrolysis of these lipoproteins as well as the uptake of free fatty acids into various tissues [9, 32, 33]. Due to the important role of this enzyme in regulating lipoprotein metabolism and tissue specific utilization of lipids, its activity is carefully regulated in both the fasting and the fed state by various interacting proteins such as members of the ANGPTL protein family [7–9, 16]. In the fasting state ANGPTL4 expression is induced through the action of the PPAR gamma transcription factor that is induced by fatty acids released from TG hydrolysis [7]. During the fasting condition LPL activity is inhibited in white adipose tissue and activated in cardiac and skeletal muscles to increase TG hydrolysis in these tissues [16]. Similarly, ANGPTL3 and 8 play a critical role in regulating the TG plasma level particularly under feeding condition; where more of the TG is directed toward the adipose tissue for storage [16]. Their inhibition of LPL activity occurs as a result of their interaction. Studies however have shown that unlike ANGPTL4 and 8, ANGPTL3 is not nutritionally regulated [12]. Therefore, it has been suggested that ANGPTL3 activity is rather regulated through ANGPTL8 that is in turn regulated by nutrition [12]. Our data shows that the three ANGPTL proteins studied are increased in obesity, highlighting the possibility that these proteins might be involved and perhaps responsible for the increased TG plasma levels in obese and T2D subjects. Further follow up studies into their mechanism of action are ought to uncover their pathophysiological role in these diseases.

Due to their role in regulating plasma lipid content and nutrient sensing, therapeutic modulation of the activity of ANGPTL3, 4 and 8 is currently under considerable investigation as potential targets in the treatment of dyslipidemia [7–9]. For example, monoclonal antibodies targeting ANGPTL4 have been used in animal models and resulted in reduced TG level as well as higher LPL activity [7]. Similarly ANGPTL3 specific antibodies were responsible for reduced plasma TG in both mice and monkeys [34–36]. A recent study, by Fu et al. showed that targeting ANGPTL8 using monoclonal antibodies in mice resulted in reduced TG level and reduced LPL activity in both heart and skeletal muscles but not in white adipose tissue [37]. Our data further highlights the potential differential interaction between ANGPTL8 with ANGPTL3 and 4. This provides more knowledge about their role and how they are affected by various metabolic diseases leading to better understanding of their biological significance as well as improving their drug targeting abilities. Our findings that ANGPTL4 and 8 positively associate with each other may be contradictory as they are induced at different nutritional states; fasting and feeding respectively. However, this could be partially explained by a recent model based on animal data proposed by Zhang called ANGPTL3-4-8 model [16]. In this model, it’s proposed that these three proteins regulate LPL in a tissue specific manner according to the feeding status. In this model, it has been suggested that ANGPTL4 is induced in the fasting state to inhibit LPL in WAT directing TG to cardiac and skeletal muscles [16]. Whereas; during feeding, ANGPTL8 is induced, which acts through the interaction with ANGPTL3 to inhibit LPL activity in cardiac and skeletal muscles directing TG to adipose tissues for storage.

One of the main limitations of this study is the cross sectional design, which does not allow us to establish the biological role of the studied proteins in the development of diabetes. However, due to their well-known function in lipid regulation, it is assumed that their increase in obesity will lead to dyslipidemia that will contribute to the increased insulin resistance and eventually lead to the development of metabolic diseases like T2D. Another limitation was the use of gene expression data from adipose tissue. It would have been more appropriate to study the protein expression instead of the gene expression, but due to the scarcity of the adipose tissues that were extracted from volunteers, only gene expression was possible.

## Conclusions

In conclusion, we have comprehensively investigated the association between ANGPTL3, 4 and 8 in obesity and T2D in plasma and adipose tissue showing for the first time that ANGPTL 4 and 8 are increased in human subjects with T2D compared to non-diabetic subjects. ANGPTL3 on the other hand was only increased in circulation but not adipose tissues. Obese subjects had higher level of ANGPTL3, 4 and 8 compared to non-obese in the non-diabetic subjects. However, their expression in T2D subjects was not affected by obesity. ANGPTL3 was associated with ANGPTL8 in the non-diabetic subjects, whereas, ANGPTL4 was associated with ANGPTL8 in the obese subjects regardless of their diabetes status. Finally, ANGPTL3, 4 and 8 are impotent regulators of lipid metabolism and they offer potential therapeutic targets for lowering level of plasma lipids.
